# Letter from Edmund Andrews, M.D., on Venereal Diseases and Prostitution under the License System of Europe

**Published:** 1867-10

**Authors:** Edmund Andrews

**Affiliations:** Paris


### VENEREAL DISEASES AND PROSTITUTION UNDER THE LICENSE SYSTEM OF EUROPE.—THE LAWS, STATISTICS, AND RESULTS OF THE SYSTEM.

PARIS, August 8, 1867.

As American cities increase in population, the evil of prostitution becomes more and more manifest. The question whether it shall continue to be prohibited by law and contended against by the police, or be tolerated as unavoidable and placed under licenses and regulations, is exciting thought and perplexity in the minds of many of our wisest men.

With the view of throwing light on this difficult question, I have expended much labor in obtaining the facts and statistics of the principal cities, both of Europe and America, a work by no means easy of accomplishment, but I have succeeded so far that I think the question may be decided in the light of the facts which I have accumulated.

I am indebted to the following sources for my information, *viz.:*—The Chief of Police in Chicago, the Chief of Police in Philadelphia, the Chief of Police in New York, the Chief of Police in Liverpool, the Board of Health, New York; the Director of the Administration of Public Assistance, Paris; the officers and records of the hospitals of Chicago, Philadelphia, New York, Liverpool, London, and Paris; the police reports of London; and the writings of Parent-Duchatelet, Trébuchet and Poirat-Duval. I am under especial obligations to Capt. Kennedy, Chief of Police in New York, to the Chief of Police in Liverpool, and to the Administration of Public Assistance in Paris, for their full explanations of their departments, and for the records and statistics of their offices.

The practical question is this:—Shall we recognize prostitution as an unavoidable evil, and try to regulate and ameliorate it by a system of licenses and regulations; or shall we put it on the same footing as other crimes, and endeavor to confront it with direct opposition?

The usual arguments, on both sides, are these, *viz.:*—

1. Prostitution has always existed. It is practically impossible to extinguish it by police regulations. It is better, therefore, to recognize what is unavoidable, and put it under sanitary control, with the view of limiting the spread of venereal diseases. It is further urged, that the patrons of prostitutes are not the only sufferers, but that they marry innocent parties and transmit to their wives and children the diseases which they obtained in the houses of infamy. Now, a weekly inspection of the prostitutes, and putting the diseased under treatment, may be expected to greatly diminish the proportion of such cases among them, and, consequently, to diminish the infection carried from them to innocent parties. It is further contended, that by putting these women under such a system of regulation, they can be prevented from exhibiting themselves and soliciting custom on the street; that their houses, being always under surveillance, can be rendered more orderly; and, finally, in a system of licensed houses, the police know better where to look for thieves and other malefactors whom justice demands, because they always haunt these dens.

On the other side of the question, it is replied, that the legalizing of a crime is abhorrent to the moral sense of community. If it is true that it is impossible wholly to extinguish prostitution by legal means, the same may be said of theft and murder, and all other crimes. No doubt, the thieves could be made more orderly if they were registered and licensed. It is feared also that the effect of giving prostitution a regular license and the sanction of law, would be to throw around it a sort of respectability which would increase its evil influence. It is also feared that the effect of undertaking to keep these women free from disease for the benefit of their patrons, would not be an unmixed good. The question arises, whether the fact that government, with a corps of surgeons, is steadily at work examining and removing diseased women from the houses, will not have the effect to cause the unwary to suppose these places to be safe resorts, and thus to greatly increase their patronage, so that the increased prostitution would bring up the sum total of disease in the community to as great a figure as before? If the disease is to be nearly the same and the prostitution to be increased by the system, then the result would be an injury, beyond question.

It will be noticed that all these arguments, *pro* and *con*, are theoretical. What we want is the facts. What is the toleration system? What are its results? Are the cities where it exists more moral, or more free from venereal disease, than where it does not? The facts below are the answer.

The outlines of the system are these:—The laws announce to the prostitutes that, although their crimes are worthy of punishment, the practical difficulties of the subject have determined the authorities to tolerate prostitution under certain conditions and regulations. These conditions are usually as follows:—

1st. Every woman proposing to act as a prostitute shall, under penalty of imprisonment for refusal, register her name at the police head-quarters, in the book provided for the purpose, and shall only exercise her vocation in the apartment or house permitted by her license.

2d. She shall never walk the streets in gay dresses, nor in any manner, by look, or word, or gesture, solicit patronage in public places. In some cities they are prohibited from going to theatres, concerts, balls, or other places of public amusement; but in Paris they have immense dance-houses, where they fully revenge themselves for their restrictions on the street.

3d. She shall, at all times, keep a proper array of syringes and sponges, and, after each act of coition, shall thoroughly wash out her vagina without delay.

4th. The houses and apartments shall be subject to police inspection at all hours.

5th. The public women are divided into two classes, the *filles de chambre* and *filles de maison.* The former take a license to live by themselves in private apartments; the latter go into houses of prostitution, kept by women who have absolute authority over them, and frequently treat them with great cruelty.

6th. The *filles de chambre* shall appear at the appointed place once in two weeks, and the *filles de maison* every week, for surgical examination. Here, a staff of surgeons takes them in hand, and examines the vagina, the rectum, and the mouth. Those found diseased are sent to a prison-hospital, where they are strictly confined until they are cured. Those not diseased, are furnished with a certificate of health, with the date of the examination. Those that fail to present themselves for examination are sought out and punished.

There are numerous other regulations for their government, but the above are the main points in most of the cities where the system exists. The next question is, what are the results? Have the cities thus regulated fewer prostitutes than others? Have they less venereal disease than others?

To answer these questions, I have, with much labor, gathered the material for the following tabular statements. I place in the first table the cities which have in operation the toleration system, with the number of registered prostitutes in each. The second table contains those which have not this system. The third shows the proportion of venereal disease in the hospitals of the non-licensing cities, which is compared with that of Paris.

*Table showing the proportion of Registered Prostitutes to the whole population, in cities which adopt the License System.*

| | | | | |
|---|---|---|---|---|
| Paris, | 1 | Prostitute to | 281 | Inhabitants. |
| Brussels, | 1 | " | 435 | " |
| Berlin, | 1 | " | 754 | " |
| Copenhagen, | 1 | " | 564 | " |
| Hamburg, | 1 | " | 359 | " |
| La Haye, | 1 | " | 750 | " |
| Rotterdam, | 1 | " | 267 | " |
| Amsterdam, | 1 | " | 286 | " |
| Turin, | 1 | " | 193 | " |
| Bordeaux, | 1 | " | 270 | " |
| Brest, | 1 | " | 159 | " |
| Lyons, | 1 | " | 424 | " |
| Marseilles, | 1 | " | 286 | " |
| Nantz, | 1 | " | 378 | " |
| Strasbourg, | 1 | " | 281 | " |
| Algiers, | 1 | " | 102 | " |
| Average, | 1 | Prostitute to | 362 | inhabitants. |

This table, I have formed by combining the statistics found in the supplement to Parent-Duchatelet's great work, third edition, issued in 1857. The figures are given on the authority of the Chief of the Sanitary Bureau and of the Chief of the Bureau of Police, of Paris.

*Table showing the proportions of Prostitutes to the whole population in cities which do not adopt the License System.*

| | | | | |
|---|---|---|---|---|
| Chicago, | 1 | Prostitute to | 230 | Inhabitants. |
| New York and Suburbs, | 1 | " | 518 | " |
| London and Suburbs, | 1 | " | 544 | " |
| English great seaports, Liverpool, Bristol, & Plymouth, | 1 | " | 193 | " |
| English pleasure towns as Brighton, Bath, etc. | 1 | " | 244 | " |
| English towns depending on agricult'l districts, as Ipswich. | 1 | " | 309 | " |
| Seats of English Cotton & Linen M'f'res as Manchester, etc., | 1 | " | 557 | " |
| Seats of English Woollen Manufactures, as Leeds, etc., | 1 | " | 654 | " |
| Seats of English Mix'd Manufactures, as Norwich, | 1 | " | 478 | " |
| Seats of English Iron M'f'es, as Birmingham, Sheffield, etc., | 1 | " | 709 | " |
| Glasgow, | 1 | " | 394 | " |
| Madrid, | 1 | " | 270 | " |

Average, 1 Prostitute to 425 Inhabitants.

From which it appears that the proportion of prostitutes to the population is about 17 per cent greater in cities which adopt the license system, than in those which do not.

The great aim of the license system, however, is to diminish the scourge of venereal disease in the community at large. It is important, therefore, to enquire what the per cent of venereal cases is in the two classes of cities. The difficulties of this investigation are very great, but I have adopted for a standard of comparison the proportion found in the civil hospitals of the various cities, excluding from the comparison the military hospitals and the prisons. This method will be as fair for one class as for the other. I have succeeded in obtaining the results in six non-licensing cities, and in one licensing city, *viz.*, Paris. In the other European cities, I have been unable to get the statistics with sufficient accuracy to entitle them to places in the tables.

*Table showing the proportion of Venereal Patients to all kinds treated in civil hospitals.*

| Cities | Venereal Cases. | Total Cases of all Kinds. |
|---|---|---|
| Chicago, | 580 | 4,147 |
| Philadelphia, | 1,127 | 12,292 |
| New York, | 1,833 | 23,314 |
| Liverpool, | 2,073 | 63,074* |
| Manchester, | 3,500 | 75,000* |
| London, | 3,357 | 31,264 |
| Totals, | 12,470 | 209,091 |

Or one venereal case to about 16¾ of all kinds.

In Paris, which has the license system, I find in the same class of hospitals, as follows:—Venereal cases, 5276; cases of all kinds, 84,267, or 1 venereal case to about 16 of all kinds. It appears, therefore, that there is a trifle more venereal disease in Paris than in the average of cities which have no license system, in spite of the weekly examination of the strumpets.

This result, which at first glance seems so surprising, is readily accounted for, if we look a little further into the practical working of the system. The weekly inspection of the strumpets, under the license system, greatly diminishes the *proportion* of disease among the strumpets themselves, but this very fact

* The figures of Manchester and Liverpool, both of venereal and of other diseases, appear very large, because they include the out-patients as well as the in-patients. In the other cities, there are no statistics of the out-patients.

acts as a first-class advertisement of the safety of their charms. Hence, their patronage is increased, as is shown by the increased numbers, and this augmented number of risks goes far to balance the diminished danger of each single copulation. In non-licensing cities, the fear and disgust of diseased strumpets acts on many minds as a strong deterring influence.

Again, it is a great mistake to suppose that the license system ever effects a complete regulation of this evil. The Chief of Police in Paris shows that in every city where the plan is attempted, there is a large class of prostitutes, called *insoumis*, or *femmes libres*, who prefer their liberty to police restrictions, and in spite of law carry on their trade clandestinely. Having no medical inspection, they are as much diseased as the prostitutes of any other country. There are no more thorough police corps in the world than those of Paris and Berlin, yet in these cities a large portion of the habitual prostitutes are neither registered nor regulated. At the prison hospital of St. Lazare, where all diseased prostitutes are sent for cure, the surgeon informed me that more than half the patients are *femmes libres*—unregistered, illegal prostitutes—captured by the police in the exercise of their profession. In Berlin, in 1857, the number of these clandestine prostitutes was reckoned as far exceeding that of the registered ones. (Parent-Duchatelet's work, vol. ii., p. 675.) If, therefore, it is true that no police has ever been able to suppress prostitution, it is equally true that the best police in the world can neither regulate it nor even register it with anything like completeness. The reasons of this failure are as follows:—

1st. The invincible determination of many of these women to preserve their liberty, and to avoid the restrictions of police regulations. This is human nature the world over, and there is no police which can compress it out of existence.

2d. The police often know to a moral certainty that a woman is living by prostitution, but if she it quiet and orderly, and if there is not clear legal proof of her actions, a seizure of her person, and a consigning of her by force to the infamy of a house of prostitution, excites a degree of popular resentment which neither the police of Paris nor any other city are willing to brave.

3d. The same remarks are applicable to clandestine prostitutes of orderly behavior, who make a fair pretence of living by some legitimate business.

4th. There is a class of prostitutes, who are pursuing their trade under the roofs of their parents, often with their approval. If they are not disorderly, they have a sort of quasi respectability, and have still a chance to marry and reform, which they occasionally do. Practically, the magistrates, though knowing their character and probable ultimate destiny, shrink from tearing them from the parental protection, and from sundering the last tie which connects them to the possibility of a virtuous life.

5th. So far as I can ascertain, no effort is made to register the mistresses of men who keep loose women for their own exclusive use.

6th. No police pretends to supervise that indefinite mass of licentiousness which prevails *con amore* among the people, and is a matter of passion and not of pecuniary consideration.

From these various facts, it results that the European officers of justice are unable to reach more than a small portion of the sources of venereal contagion. They prevent a part of the disease of the registered prostitutes, but the great tide of clandestine licentiousness flows past them unchecked, and saturated with syphilitic infection. I think that the statistics plainly show three things:—

1. The license system never succeeds in putting anything like the whole number even of professional prostitutes under regulation, much less the semi-professional ones.

2. It does not diminish the sum total of venereal disease in community, but only the relative risk per copulation.

3. It increases the amount of public prostitution, by giving an impression of the safety of the inspected brothels, and thus affording them the very best possible advertisement. Meanwhile, it never fully frees them from infection, and the increased number keeps up the sum total of disease.

There are, however, some minor advantages gained. Thus, the prostitutes are kept, in a great measure, from open solicitations on the streets, so that cities under this plan present a greater external appearance of decency than others, even though they may be more corrupt in fact. Also, by having the brothels under constant observation, the police prevent many disorders, and when thieves or other malefactors are to be sought for, these houses are excellent man-traps, in which to catch them. This consideration, I find, always weighs strongly among police officers, in favor of the system, as might be expected from the nature of their duties.

On the whole, it does not appear that the license system is worth transporting to America in its present form. It increases the licentiousness without diminishing the sum total of venereal disease, and this fact is enough to condemn it. I think, however, that some improvement must be made in our present American plan, or rather our *no plan at all*, of managing this evil, but I think we shall be obliged to devise it ourselves, for there is no system in Europe worth copying.

There are some curious developments of human nature in Parisian licentiousness. The strumpets often have a high opinion of themselves, and are even very pious. Here are specimens of their petitions to the Prefect of Police. One commences thus:—

"*Monsieur le Prefet:*—

"Being incapable of labor, in consequence of a double hernia and other grave maladies, it is not for the gratification of my passions, nor from bad habits, that I have for ten years been enrolled in your office (as a prostitute). The testimony from all my neighborhood, Monsieur le Prefet, will prove to you that I have, to some extent, effaced by my *morality*, *my decency*, and the *regularity of my conduct*, the degredation of my condition."

An old woman, aged 82, applies in the following terms, for permission for her daughter and granddaughter to open a new house of prostitution:—

"*Monsieur le Prefet:*—

"At the age of 82 years, and the mother of a numerous family, I implore, Monsieur le Prefet, your aid and protection. You who are the father of the poor, the support of the widow and of the orphan, the stay of the afflicted, and the refuge of the unfortunate, will certainly not refuse my request. At an age so advanced, feeling myself near the time when I must render up my soul to God, and appear before my Creator, it is my duty to provide for the wants of my children, and to transmit to them my means of subsistence." Then follows her request for permission to establish a brothel, under the charge of her daughter.

Another woman petitions for leave to open a new house, promising the Prefect that she will keep in it only "the most distinguished women," who will attract men of such irreproachable character, that "tranquility, order, faithfulness, and healthfulness will be the inevitable result." Moreover, she declares that the splendor of the house and its furniture will be such, that Paris will glory in it.

Another commences her petition as follows:—

"*Monsieur le Prefet:*—

"I have been a registered woman for ten years, and have lived in my own apartments (*fille de chambre*). Feeling bound in honor to preserve intact the reputation of *honesty and delicacy* which I have acquired in my neighborhood, I am forced, in order to fulfil *sacred engagements and to pay some debts of honor*," etc., etc.

It is evident that the Parisian prostitutes preserve their full share of the power of gasconade. The depth of degradation to which these poor women are driven by their paramours is almost inconceivable. The chief-surgeon of the prison hospital of St. Lazare informed me that one woman sometimes sustains forty connections in twenty-four hours, working day and night, to use his own comparison, *comme une machine a coudre a vapeur* (like a steam sewing machine). He states, also, that the men of the Parisian hells have introduced and brought into full vogue, the Oriental practice of copulating with these women *per anum*, instead of *per vaginam.* As an illustration, he showed me a considerable number of women with a peculiar hypertrophy of the anterior border of the anus, produced by this practice. Several of them had the circumference of the rectum studded with chancroidal ulcers, as the result. What is still more incredible, is that these depraved beasts, driven to their wit's end for new measures to excite their jaded passions, frequently copulate *per os faciis*, so that primary sores are now frequent on the lips and in the interior of the mouth. He showed me several patients thus affected, remarking with French vivacity, respecting one of them who had a large chancre on her lip, "elle a joué la clarinette avec un penis d'un homme pour instrument" (she has been playing the clarionet, with a man's penis for an instrument). As a consequence, the the examining surgeons are obliged to inspect the mouth, the vagina, and the anus, before they can give a certificate of soundness.

If, in relating the above facts, I have offended any one's delicacy, I am sorry for it. I deem it important at the present time, when many are proposing to adopt in America the French system, to state the whole truth, that we may know what style of vice flourishes under that system, and judge for ourselves whether it is smaller in its magnitude, or any less revolting in form than that which we already have in our cities.

As the present continental system is a failure, it brings back the question with perplexing emphasis, What is to be done? Chicago, like all cities where there is a large floating population, has a large share of the social evil, and it does not seem possible to rest in our present condition. The method now in use with us, is to make no effort to break up the brothels, but simply to tolerate them and use them as a means of raising money for the city treasury. For this purpose, the police are sent at irregular intervals to make a "descent" on the houses, when they bring up the inmates to pay a fine. They pay it, of course, as if it were a regular tax, and return at once to their business. It is not expected to break them up by this method.

The plan amounts to a kind of license for fees, but the money is collected in a vexatious and irregular manner that is not creditable to a good government. It seems to me that we should not proceed suddenly to a new and complex system of management, but advance step by step, as experience shows us the road. Probably it would be best in the first place tò abandon this system of irregular fines, and allow the brothels a tacit toleration, not in return for fines, but in consideration of strict quietness and good order.

Another step that should be taken is, to make as complete a registry as possible both of houses and inmates. Possibly some new legislation may be required to give the police department additional powers for this purpose. Capt. Kennedy, of New York, takes an accurate census of the prostitutes once a year. The Chicago police keep a careful register of the houses, but not of the persons. It seems necessary to empower the police to make a register of the *persons*. The persons thus registered should not be molested so long as they observe good order, and do not parade themselves in public places; and the fines and imprisonments should be reserved for those who evade the registry or are guilty of violating the rules laid down for their regulation. Of course, the police should have access to the houses at all hours.

As to the diseases, it appears not worth while to tax the whole community for a costly array of hospitals and surgeons for the benefit of the patrons of the brothels. The system in Paris shows no success in extirpating syphilis from the community. For the present, at least, it would seem best to leave the disease as one of the punishments of licentiousness, and let those who have it pay their own doctors to be cured. After having advanced thus far, experience would readily show what other steps should be taken. To sum up, therefore, my proposition is this:—

1. Abandon the infliction of irregular fines.
2. Have a careful registry of brothels and prostitutes.
3. Those women who register themselves should have a tacit toleration, so long as they observe good order and keep the rules laid down to them.

4. Those who do not register, and those who violate the rules, should be visited sharply with fines and imprisonments.

5. Have the houses under close observation by the police.

EDMUND ANDREWS, M.D.

NOTE 1.—I have in my possession a copy of a report of a committee of the Harveian Society, of London, on the subject of prostitution. It contains some statistics as to the proportion of venereal patients in many English hospitals, and I hoped at first to derive great assistance in my investigations from this document. I found, however, on personal investigation at some of the hospitals mentioned, that the report contained enormous errors, not intentionally, but, apparently, from want of rigid scrutiny of its sources of information. This unfortunate fact throws suspicion upon the reliability of its whole statistics, and as I had no means of completely separating its truths from its errors, I was reluctantly obliged to omit its figures from my tables, except where I could confirm them from other sources. I understand that the same society proposes to lay its information before theInternational Congress at Paris, which is to be greatly regretted, if they do not correct it. This report was prepared with a view of making a startling impression on the public, as to the prevalence of venereal disease in England, in order to agitate for the passage of a law on the subject. With this view, the committee seems to have sought for information adapted to the purpose, and to have been deceived by incorrect statements. Wherever I was able to test the figures, I usually found them to greatly exaggerate the amount of venereal disease. The French journals are already quoting this report, and making many exclamations of horror at the *licentiousness of the English!*

NOTE 2—There is a recent act of Parliament in England, which, for the purpose of protecting the soldiers and sailors, has designated twelve towns—the chief garrison and naval towns of the kingdom—in which the police are authorized to arrest strumpets for examination, and confine the diseased until they are cured. It is claimed that this act greatly diminishes the disease in the army and navy. I have had no opportunity to verify the statement, but I have little doubt of its truth. European soldiers and sailors, as a class, consort freely with *public* prostitutes, and much less with *clandestine* ones. They take the risk of disease without disgust or hesitation. Hence, I have no doubt that a regular inspection and treatment of the inmates of the brothels would diminish the disease among these men, even though the rest of the community was the worse for the system. There are no statistics, that I can find, showing the effect of the law on the disease among the citizens of these towns.

